# Young Adults With Type 1 Diabetes and Their Perspectives on Diabetes-Related Social Media: Qualitative Study

**DOI:** 10.2196/69243

**Published:** 2025-06-25

**Authors:** Tara Maxwell, Lillian Branka, Noa Asher, Persis Commissariat, Lori Laffel

**Affiliations:** 1 Division of Endocrinology & Diabetes, Children's Hospital of Philadelphia, 2716 South Street, Roberts Building, 14th Foor, Philadelphia, PA, 19146, United States, 1 2155903174; 2 Department of Pediatrics, Perelman School of Medicine, University of Pennsylvania, Philadelphia, PA, United States; 3 Section on Clinical, Behavioral and Outcomes Research, Joslin Diabetes Center, Boston, MA, United States; 4 Harvard Medical School, Boston, MA, United States

**Keywords:** diabetes mellitus type 1, social media, young adult, self care, qualitative

## Abstract

**Background:**

Young adults with type 1 diabetes (T1D) often struggle with self-management and achieving target glycemic control, and thus, may benefit from additional support during this challenging developmental life stage. They are also some of the highest users of social media (SM), which may have some benefits to young people with T1D.

**Objective:**

Given the potential of SM support for people with diabetes, we sought to use qualitative methods to explore the perceptions of diabetes SM posts to influence self-care and emotional state of young adults with T1D.

**Methods:**

A series of Instagram (Meta) posts were selected by a multidisciplinary team of T1D experts. Young adults aged 18‐25 years with T1D duration of 1 year or more were recruited from the clinic to participate in a 60-minute semistructured videoconferencing interview. First, they were queried about their SM use in general and specific to diabetes. Next, they reviewed 10 posts with the interviewer. For each post, perceptions and reactions were queried. Participants were asked about each post’s impact on their emotional state and potential influence on diabetes self-care. Finally, they were asked to comment on what the posts emphasized and how they felt after viewing the posts. Interviews were transcribed and coded using thematic analysis. The participants’ diabetes management information was extracted from the electronic health record.

**Results:**

There were 26 young adults who completed the study. Their mean (SD) age was 22.6 (SD 2.0) years, T1D duration 12.6 (SD 5.9) years, and glycated hemoglobin (HbA_1c_) 7.6 (SD 1.2%). In this sample, 65.3 were female and 84.6% White. All were using continuous glucose monitors (CGMs) and 80.7% used insulin pumps, 71.4% of which were hybrid closed loop. All participants used SM at least once daily, but most only sometimes or rarely used SM to access diabetes content and rarely or never posted diabetes content themselves. Major themes arising from the interviews centered on the potential for the young adult to connect emotionally through SM, which could be either positive or negative. Overall, for young adults with T1D, SM served to (1) highlight the existence of a community of people with T1D, (2) be a source of new diabetes information, (3) potentially influence diabetes self-management, (4) potentially influence emotional state, and (5) be appealing to the T1D community when the posts possessed certain characteristics (eg, medical accuracy, aesthetically appealing presentation of content).

**Conclusions:**

SM has the potential to help young adults with T1D feel a sense of community, seek and share objective and subjective thoughts and feelings about diabetes, motivate diabetes self-care, and positively affect emotional state. However, it may also have the potential to demotivate self-care and exacerbate negative emotional state with regards to diabetes.

## Introduction

Young adulthood, generally defined as ages 18‐25 years, is a time of major transition with increasing developmental autonomy, which can make diabetes self-management more challenging for those people with T1D[1]. Young adults struggle with attainment of target glycemic control, even in the current era of advanced diabetes technologies that generally reduce the burdens of T1D self-care [2]. Young adults often begin to display clinical evidence of long-term renal and ophthalmologic complications [3], and frequently experience lower health-related quality of life [4]. Diabetes distress is also common in this group, often attributed to stigma, the rigors of day-to-day diabetes self-care, and financial worries [5,6]. Given these complexities, young adults may benefit from more support during this time of increased transitions and competing obligations. To meet this need for support, creative means have been explored [7-10], and especially following the COVID-19 pandemic, which emphasize use of the virtual and digital environments. One important aspect of this environment is social media (SM).

Given that young adults are some of the highest users of SM [11], it follows that this modality may be a resource for peer support, a creative means to overcome and promote problem solving for to their diabetes challenges, and source for new information. Furthermore, there are known benefits to online support for people with diabetes [12]. One study comparing the SM use of young adults with T1D with those with inflammatory bowel disease showed that those people with T1D were more likely to use SM to share content about their disease, engage in peer support groups, and seek information [13]. Researchers have begun to explore the relationship young adults with T1D have with SM, describe its use to access diabetes medications and supplies [14], and have used SM to recruit young adults with T1D for studies [15].

Investigators have examined existing T1D content created by people with T1D on social media to better understand the virtual diabetes environment.. For example, Tenderich et al [16] used qualitative methods to describe T1D-related content on various SM platforms, having 6 main themes: humor, diabetic pride, getting personal with diabetes tech, tips and tricks, building community, and venting . Holtz and Kanthawala also qualitatively reviewed SM posts, focusing on Instagram (Meta) posts about T1D, finding more positive than negative sentiment in posts, and that self-disclosure of diabetes was associated with increased post engagement [17].

There remains a need to understand the perceived impact of diabetes-specific SM from perspectives of the people with diabetes who view it. This information may be beneficial for clinicians, researchers, and other stakeholders to reach and support young adults with T1D through SM posts in a way that is relevant and useful. In this study, we aimed to use qualitative methods to learn young adult’s perceptions of various SM posts, and if how they believed these posts may impact their diabetes self-care and emotional state.

## Methods

### Study Design

A multidisciplinary team (pediatric endocrinologists, psychologists, diabetes educators, and nurse practitioners) determined study design, produced interview questions, and selected relevant SM posts with iterative review through meetings and discussion with young people in the target age group. First, it was decided that posts would be selected from Instagram. Instagram has been reported as the most frequently used platform in those aged 18‐29 years [18]. Next, research staff members reviewed posts by searching #type1diabetes, #t1d, and related hashtag or accounts to capture themes described by Tenderich et al [16]: humor, diabetic pride, getting personal with diabetes tech, tips and tricks, building community, and venting. Posts were reviewed by the study team, and of the approximately 50 initial posts reviewed, 10 were selected to be discussed in interviews with research participants. Finally, an interview guide was developed to explore participants’ reactions to viewing the SM posts, their engagement with content, and potential of the post changing behaviors or emotions.

Eligible young adults were ages 18 to 25 years old, with T1D duration of at least 1 year, and fluent in English. Those with significant developmental, cognitive, or psychiatric disorders (including inpatient psychiatric admissions from the past 6 months) were ineligible. Use of SM use was not a requirement to participate. All eligible participants were seen in the Pediatric, Adolescent, and Young Adult Section of the Joslin Diabetes Center, and recruited through provider referral.

Following provider referral, a study team member contacted interested young adults to schedule a one-time video call over the Health Insurance Portability and Accountability Act (HIPAA)-compliant Microsoft Teams platform. Informed consent was obtained, and electronic signature was provided via REDCap (Research Electronic Data Capture, Vanderbilt University) [19,20] before the start of any study procedures. Consent also detailed permission to access each participant’s electronic health record (EHR) to pull specific demographic and diabetes management information (eg, age, sex, diabetes duration, diabetes technology use, and glycated hemoglobin [HbA_1c_]).

Participants engaged in a one-on-one interview with study staff in which the 10 selected posts were viewed and participant experiences, perceptions, and reactions were queried. To reduce likelihood that interpretation of posts could be affected by their placement in the interview, staff members used 4 distinct collections of the same 10 posts in different orders. The semistructured interview guide can be found in Textbox 1. Research staff interrogated the EHR for demographics and diabetes care characteristics as outlined above.

Textbox 1. **Interview questions**

**Basic social media use questions**

- How often do you use social media?
- How often do you use social media to view others’ experiences living with diabetes?
- How often do you use social media to share your experiences living with diabetes?

**Questions asked for each post**

- Can you tell me what you see in this post? What are your initial reactions to this post? What stands out to you about this post? Why?
- How does this post make you feel?
- Do you recognize the person/account that posted this? (If yes) from where?
- Would you “Like” this post? Why or why not?
- Would you share this post with others? Why or why not? (If yes) how? With whom?
- How might this post impact the way you manage your diabetes after you see it? Why/Why not (if appropriate)? How might this post change your willingness to take care of your diabetes? Why/Why not (if appropriate)?

**Concluding questions**

- Now you’ve seen that each of these posts includes the post itself, the account that posted it, captions, likes, and hashtags. What do you feel like you focused on the most when viewing these posts? Why?
- Now that you have spent some time looking at these posts, how do you feel about living with diabetes?
- How does this compare to how you felt before looking at these posts? Is there anything else you’d like to share with us about diabetes and social media?

An inductive approach to thematic analysis [21] was used to analyze transcripts and elicit new information about young adults’ experiences with SM. The primary investigator coded all transcripts individually using NVivo (Lumivera, release 1.5.2, QSR International) to create an initial codebook; this coder had viewed and selected the SM posts. All transcripts were then double-coded by a second team member using the initial codebook; the second coder had not selected or viewed the SM posts. Coders met regularly to discuss discrepancies and revise the codebook accordingly. All coding discrepancies were discussed between coders, and disagreement was brought to a third study team member until consensus was reached [21]. Following coding, study staff further assessed codes by reviewing quotes and documenting the major distinct ideas in the data. These idea units were then further organized into key dimensions that study staff determined to be most relevant to the current study, including participants’ initial reactions to posts, knowledge gained, emotional responses, and potential impacts on self-care. Major themes around young adult’s perceptions of diabetes-specific SM posts were elicited from this focusing exercise. Basic descriptive statistics of the study sample were performed in Microsoft Excel.

### Ethical Considerations

The study protocol was approved by the Joslin Diabetes Center Committee on Human Subjects (CHS #163). Following the conclusion of the interview, participants were mailed a US $25 Visa gift card for their time. Informed consent was obtained before the start of any study procedures.

## Results

### Sample

There were 26 young adults aged 18‐25 years with T1D for at least 1 year who completed the study. Participant characteristics can be found in Table 1.

**Table 1.** Participant baseline data.

Participant characteristics (N=26)		Values
Age (years), mean (SD)		22.6 (2)
Sex, n (%)		
Female		17 (65.3)
Race, n (%)		
White		22 (84.6)
T1D^a^ duration (years), mean (SD)		12.6 (5.9)
HbA1c^b^, % (mean (SD)		7.6 (1.2)
CGM^c^ use, %, mean (SD)		
Time in range (%)		100
mean (SD)		54.5 (19.6)
Pump use, n (%)		21 (80.7)
Hybrid closed loop, n		15

a T1D: type 1 diabetes.

b HbA_1c_: hemoglobin A_1c_.

c CGM: continuous glucose monitor.

When asked about their baseline SM use, 100% of participants stated they used SM daily or more. When asked how often they use SM to view others’ experiences with diabetes, the most common response was rarely (11, 42.3%), followed by sometimes (9, 34.6%), often (3, 11.5%), daily or more (2, 7.7%), and never (1, 3.8%). When asked how often they use SM to share their own experiences with diabetes, the majority said rarely (14, 53.8%) or never (9, 34.6%). There was one participant each who said daily or more, often, or sometimes (1, 3.8%).

### Themes

Major themes that arose from the data centered around SM as a platform for a range of connections with T1D from positive to negative, as participants discussed how T1D SM could (1) highlight the existence of a community of people with T1D; (2) be a source of new information about T1D; (3) potentially influence diabetes self-management; (4) potentially impact emotional state; and (5) possess aesthetic appeal and relevancy to people with type 1 diabetes.

SM can highlight the existence of the T1D community. The majority of participants endorsed feeling positive toward SM posts that reminded that there are many others with T1D with similar experiences. With this realization, some reported feeling less alone.

It’s kind of like an instant relation. You can say, ”Hey, I’ve been there.” You see that somebody else is going through it. It’s not just you. Somebody else is having that same problem, and they’re just as upset about it.

[24-year-old man]

However, others experienced negative reactions to posts highlighting the T1D community when they lacked peer support for T1D, describing themselves as feeling jealous and more isolated upon viewing.

I was kind of jealous, actually, because I myself don’t have a lot of friends with diabetes. You know, I don’t really interact with somebody else with diabetes all that often, so I was like a little jealous, like, ‘Oh, look at these people. They’re hanging out together, and they all share this thing’ and I kind of wish I had that.

[21-year-old woman]

SM can provide new information about people with T1D and management. SM can be a source of knowledge for young people with T1D, providing tips and tricks for diabetes management. In particular, some participants posited that certain SM posts could contain helpful knowledge for those new to diabetes.

I have only had diabetes for like two years at this point, so I’m still learning a lot, and I did not know that it would be helpful to change your pump site earlier in the day than at night, so it’s good for me to see it.

[24-year-old woman]

Some young people felt more motivated to seek out more education and ask specific questions about their diabetes to their medical team.

Maybe I’d ask my doctor about like what a pump break would really mean and how I would be able to do that…because like going back to syringes is a whole, another thing, but yeah I would probably be inclined to ask about it.

[21-year-old woman]

Participants also pointed out that posts about T1D could help raise awareness and understanding in the general public.

I think anybody could benefit from this information, not just, you know, diabetics because, you know, the more people realize that [cost of insulin] is …a problem in the country, the better for everybody.

[23-year-old man]

SM can potentially influence diabetes management. Young adults reported a variety of potential influences on T1D management, ranging from positive to negative impact. Some participants noted that a SM post could serve as a helpful reminder to follow through on their diabetes care, and some also reported that the posts triggered them to think about planning for future, upcoming diabetes care tasks.

It might just be a reminder to, you know, change my [pump] site. Or on the earlier side, so maybe the next time I change my site it will be a little bit earlier…

[26-year-old woman]

I think I would definitely do something different for my diabetes, like, you know, instead of changing before I go to sleep, I would check earlier in the day to see if I need to change it, and then I would do that. Like I think it would help. I would pay attention more to my diabetes.

[21-year-old woman]

Many participants pointed out that seeing others engaged in diabetes care (injections, technology, etc) in a photo might make them more comfortable sharing or managing their own diabetes in public.

It might just make me a little less likely to like turn my arm to hide my CGM if someone’s taking a picture of me.

[20-year-old woman]

Some participants felt as if the T1D SM content discouraged them from performing their diabetes care.

I’m just a stubborn person, but any time someone tells me what to do and not ask, like I kind of have like a sour taste in my mouth, so when it says, ‘Immediately change your site,’ I’m like, ‘No, I’m going to… let it go for another two days.

[25-year-old man]

SM can potentially influence emotional state. Many participants enjoyed seeing people with diabetes thriving and displaying confidence on SM. They appreciated posts reminding them that they are not limited or defined by their diabetes.

There’s joy, you know, because it’s nice to see someone who is like me, experiences the same chronic illness...thriving out there with some really good fashion...and just being able to exist with the illness in the best possible way that can fit into their life.

[19-year-old man]

They expressed appreciation for posts reminding them that they are not limited nor defined by their diabetes, emphasizing a positive identity with diabetes.

But like the line in the caption where it’s like, ‘I’m strong, energetic, positive, passionate, and so much more.’ Like sometimes I forget those kinds of ways to describe myself, and I’ll be like, oh, I’m a diabetic, you know. When that’s not who I am, it’s just a part of what I am.

[21-year-old woman]

Participants felt positively about SM posts that encouraged self-compassion. These were posts that served to lighten the burden of diabetes, or to make viewers be more forgiving with themselves with regards to their diabetes care.

I think [the post] would encourage me to not be so hard on myself if I have a bad day of high blood sugars.

[20-year-old man]

Certain posts made some viewers feel worse. As stated previously, reminders that an online community of people with diabetes existed was often viewed as positive. However, posts displaying people in a group of others could make the viewers feel jealous and more isolated at times.

[It made me feel] a little jealous that they have friends that they can do this with…I mean, I don’t have anybody in my life that is also Type I diabetic, so I definitely feel like it would be nice to have friends around my same age who like to do the same stuff as me who can also share that experience of having to deal with their diabetes.

[25-year-old woman]

Some young adults pointed out they did not want to see reminders of diabetes on SM, particularly reminders of negative aspects of living with T1D.

I guess it’s like social media is where I go usually to get my mind off things like diabetes, and to not think about it, and so if I saw something like this, I’d be like oh man, like back to the real world.

[21-year-old woman]

There are opportunities to make SM more appealing or personally relevant to a young adult. Despite not being asked directly, many participants suggested characteristics of optimal T1D SM content. Participants shared that they appreciated posts that were realistic, relatable, with use of appealing aesthetics such as font and design, and possessing medical accuracy.

Participants commented on the aesthetic appeal of posts, noting that the framing of the information about diabetes matters.

It’s just a cool photo. It’s a good picture, and it’s like a cool way of being a diabetes post without being like super like scientific.

[25-year-old woman]

I’d be more interested in seeing what [the post] had to say if it was a bit more visually aesthetically pleasing.

[23-year-old woman]

They did not appreciate posts that oversimplified life with diabetes, that tried to be dramatic or disingenuous, and those that attempted to garner pity for having diabetes.

It makes me a little annoyed because they’re not really acknowledging like the hardness of having diabetes. It feels like someone who doesn’t really understand …[and] it is just trying to be encouraging without really acknowledging how hard it is to have diabetes.

[20-year-old woman]

Some participants expressed opinions about medical accuracy of diabetes-focused SM posts.

..this is what my doctors say, too, so it seems pretty legit.

[25-year-old woman]

…I don’t like the idea that like someone might see [the post] and think, ‘Oh, I’m just going to go on a pump break without telling my doctor’,

[22-year-old man]

Finally, some pointed out that certain content would have been more valuable soon after learning of their T1D diagnosis.

…this [post] reminds me of something I would have liked when I was newly diagnosed. I think when I was newly diagnosed, I like found comfort in these things, the idea of like making a joke out of diabetes and then finding that joke on the internet.

[22-year-old woman]

## Discussion

### Principal Findings

In this qualitative study of young adults aged 18‐25 years with T1D, we found 5 overarching themes: SM can (1) highlight the existence of a community of people with T1D, (2) be a source of new diabetes information, (3) potentially influence diabetes self-management, (4) potentially influence emotional state, and (5) be appealing to the T1D community when the posts possessed certain characteristics such as relatability, aesthetic appeal, and medical accuracy.

As far as we know, this is the first study to qualitatively describe young adult’s perspectives of diabetes-focused SM posts by querying them as they are viewing the post itself. By acknowledging the unique experiences, interpretation, and internalization of SM posts in young adults, we aim to present balanced observations that highlight the potential positives and negatives of diabetes-specific online content. Our results demonstrate the potential for SM to provide support to young people with T1D and potentially impact their well-being by fostering a sense of community and increasing access to new information about diabetes. We also describe young adult–reported positive and negative potential effects of SM on diabetes self-management and emotional state. Finally, we note suggestions for optimal T1D SM content including realism, relatability, aesthetic appeal, medical accuracy, targeting specific audiences, and avoiding dramatizing or pity regarding life with diabetes.

### Limitations

There are some limitations. First, we selected posts to conform to themes that were identified in the published literature [16,17]. In this preliminary work, we did not include posts with explicitly distressing, false, misleading, commercially-focused, or advertising content. These are all a very real part of the current SM landscape, and their influence likely needs to be assessed in future studies [22]. Next, the study was referred to as the SM study; this may have deterred those who do not regularly use SM from participating. The recruitment method, which relied heavily upon referrals from diabetes providers in clinic, could have introduced bias with a focus on young adults, for example, who were likely more attentive to their self-care. However, interviews continues until saturation in thematic responses was achieved.

As the interviews were conducted via video, there may have been an aspect of social desirability bias, where participants wanted to state observations that were pleasing to the interviewer rather than what was true. However, the interviewer was not a member of the health care team and was, in fact, a young adult with whom the participant could potentially relate. Finally, there were challenges to collecting accurate race, ethnicity, and gender identity data. Initially, the plan was to interrogate the EHR; however, we found that this information was often not provided by patients at registration and did not account for gender identity that may have varied from assigned sex at birth. We attempted to follow up with participants after the interviews but follow-up responses were very limited. In future work, we will plan to ask race, ethnicity, and gender information during study procedures rather than relying on the EHR.

### Comparison With Previous Work

The investigative plan was to probe young adult perceptions of SM in order to assess whether and how SM could provide support to young adults with T1D in their diabetes self-care and how it could affect their emotional well-being. The themes that we generated, specifically around positive influence on emotional state, appear to resonate with literature describing positive outcomes with diabetes identity incorporation [23,24] and self-compassion [25]. Posts making the viewer feel they not being limited or defined by diabetes, and those making the viewer more likely to display their diabetes technology in public, seem to promote the healthy “acceptance” illness identity dimension, which is associated with better psychological functioning and diabetes adherence [23]. Similarly, responses to posts that emphasized feeling community and commonality of diabetes diagnosis, as well as those which seemed to serve to “lighten the load” of diabetes through humor and reminders for sef-kindness, are reminiscent of interventions focusing on self-compassion, which has been associated with decreased diabetes distress [25,26].

Humor has also been demonstrated as beneficial to coping for adults with chronic illnesses [27].

The comments about the importance of accuracy of information from SM are in line with recent SM research, notably concerns about misinformation and its potential effects on young people [28], even specific to diabetes [22]. In this study, our participants seemed appropriately critical of the information in these SM posts, implying strong media literacy [29].

Our conclusion that SM can be a source of camaraderie, and knowledge for young people with T1D was in line with results from descriptive reviews of general T1D SM content itself [16,17]. In terms of comparison with any previously published descriptions of SM content by young adults with type 1 diabetes, we were unable to identify any T1D-focused studies with which to compare our observations. However, there have been some studies of young adults with other chronic illnesses that have queried their perceptions of SM. For example, a qualitative interview of young adults with cancer and their relationship with SM reported that it could be a source of community building, knowledge, and social support, but also that SM could worsen stigma, compromise privacy, and cause emotional distress based on content [30]. Our study results were similar with the exception of stigma; many participants reported appreciating the open display of wearable diabetes technologies in SM posts, with some even suggesting it may make them more comfortable taking care of their own diabetes management needs in public. This perception seems to combat, rather than worsen, stigma for young adults with T1D. Indeed, adults with type 1 and type 2 diabetes suggested advocating for their chronic disease and its management on SM to reduce diabetes stigma [31].

### Conclusions

SM has the potential to help young adults with T1D feel a sense of community, seek and share information, motivate self-care, and positively affect emotional state. However, it also has the potential to demotivate self-care and exacerbate negative emotional state with regards to diabetes. T1D SM content aimed at young adults should be realistic, relatable, aesthetically appealing, and medically accurate. It should avoid oversimplification or dramatization of life with diabetes. With engagement of the target audience of young adults, and if used judiciously, SM has the potential to positively impact young people with T1D, possibly leading improved biomedical and psychosocial outcomes.

## Abbreviations

CGM: continuous glucose monitor
CHS: Committee on Human Subjects
EHR: electronic health record
HbA_1c_: glycated hemoglobin
HIPAA: Health Insurance Portability and Accountability Act
REDCap: Research Electronic Data Capture
SM: social media
T1D: type 1 diabetes
